# Bifenthrin's Environmental Fate: An Insight Into Its Soil Sorption and Degradation Studies

**DOI:** 10.1155/jamc/8868954

**Published:** 2024-11-25

**Authors:** Jehad S. Al-Hawadi, Sara Majid, Khuram Shahzad Ahmad, Gaber E. Eldesoky, Ghulam Abbas Ashraf

**Affiliations:** ^1^Faculty of Science, Zarqa University, Zarqa 13110, Jordan; ^2^Materials and Environmental Chemistry Lab, Lab-E21, Department of Environmental Sciences, Fatima Jinnah Women University, Rawalpindi, Pakistan; ^3^Chemistry Department, College of Science, King Saud University, Riyadh 11451, Saudi Arabia; ^4^College of Environment, Hohai University, Nanjing 210098, China; ^5^New Uzbekistan University, Mustaqillik Ave. 54, Tashkent 100007, Uzbekistan

**Keywords:** batch equilibrium method, bifenthrin, insecticide, sorption

## Abstract

To fully comprehend each pesticide's behavior and interactions with soil and the environment, a thorough and nuanced analysis of each one is thought necessary. In this study, 10 randomly selected heterogeneous soil samples, each with distinct characteristics, were subjected to sorption trials as well as disintegration tests using biodegradation, hydrolysis, and photolysis. For sorption tests, the batch equilibrium approach was used, which revealed a dependence on the soils' physicochemical characteristics. Bifenthrin's distribution coefficient (K_*d*_) varied from 7.27 to 25.89 μg·ml^−1^, with *R*^2^ values varying from 0.92 to 0.99. Each soil physicochemical characteristic was associated with the various sorptive outcomes, which suggested an exothermic adsorptive reaction based on the negative thermodynamic values. The hydrolysis, soil-induced biodegradation, and photolysis processes had the shortest half-lives of bifenthrin, measuring 13.5 days, 12 days, and 121.5 days, respectively. According to these findings, bifenthrin has a moderate amount of binding and stability in soil, which makes partial decomposition of parent and daughter molecules challenging. This research advances our knowledge of bifenthrin's deteriorating processes and aids in the creation of cutting-edge strategies for ecological restoration using natural processes.

## 1. Introduction

An estimate suggests that, keeping in view of the normal scenario subject to the increase in food production by 58%–98% from year 2005 to year 2050 due to excessive and fast population growth, it is predicted to reach around roughly 9 billion people in the given time period [1, 2]. Moreover, change in dietary habits of the world due to better standard of living is also pushing the food demands to the higher extent [3]. As a consequence, the use of agrochemicals inclusive of pesticides and crop fertilizers is enhanced for coping with the needs of the world's rising population [4]. The average use of pesticide per area of crops is increased to about 71.30% from 1990 to year 2016 [5]. Excessive use of such agrochemicals posed a serious threat to pedospheric zone [6]. Additionally, such contaminants are not only limited to soil as each environmental compartment is interrelated and contamination of one zone results in serious threat to other compartments. So, the use of agrochemicals, i.e., pesticides, leaches down to the underground water resources [7] and moves into the environment through runoff and volatilization [8]. Microbial community residing in soil and their associated enzymatic activity may also be influenced in a negative way. The entry of hazardous substances and their metabolites, particularly into the food chains of humans and animals, is another issue that arises with soil contamination [9]. Many insecticides and fungicides have been disparaged for their toxic remains or residues in food chain due to their xenobiotic nature [10].

Bifenthrin (BF) (2-methylbiphenyl-yl-,3-methyl Z 1R-S cis,3), (−2 chloro-3,3 trifluoro propenyl)-2,2 dimethyl cyclopropane-carboxylate is a derivative of pyrethroid insecticide, and it is categorized as moderately toxic Class II insecticide [11, 12]. It has a low water solubility (0.10 mgL^−1^) and high K_OM_ or K_OC_ and is stable over a wide range of acidic to basic pH (i.e., 5–9.5) [13]. BF, a synthetic pyrethroid insecticide, is found in various body tissues and aquatic bodies, causing significant aquatic toxicity, making it a public concern [14].

Pesticide interacts with the soil particles by sorption (i.e., adsorption) phenomena, and hence, the fate of pesticide in the environment can only be predicted by considering the sorption properties of soil [15]. Pesticides entering soils are exposing to a variety of physical, biological, and chemical reactions altering the primeval pesticide into harmful daughter products. So, the employed pesticide undergoes sorption, hydrolytic, and photolytic reactions and degradation via microbial consortia under natural conditions [16, 17]. Ultraviolet rays of the sun are a tremendously damaging source of energy for molecular bonds, so it plays an imperative role in pesticide degradation after exposure. This source of energy is considered as an effective and imperative way of disintegrating pesticide molecules into extremely reactive daughter products. Many factors have been reported to affect degradation such as pesticide initial concentration, time of light irradiation, and solvent used [18]. In terms of biological reaction for pesticide degradation, soil microbial consortia are considered as source of certain enzymes which acts to disintegrate pesticide molecules into different products [19, 20]. Research has indicated that biogenically synthesized nanoparticles, which can be obtained from microbial or plant extracts, can function as adsorbents or catalysts to hasten the breakdown of pesticides. This presents a viable substitute for traditional degradation techniques [21]. Electrochemical methods are used to break down several pesticides such as imidacloprid that has been degraded in wastewater by electro-Fenton technology [22]. Moreover, in situ generation of reactive oxidizing species for pesticide breakdown is another electrochemical technique in the present era [23].

The entire environmental fate of the widely used pesticide BF in the chosen Asian soils has not yet been the subject of any comprehensive studies. Therefore, by performing a variety of experiments including adsorption, desorption, soil-based hydrolysis, and photodegradation, this study aimed to shed light on the fate of BF in low organic matter (OM)–heterogeneous soils. Understanding BF's sorption mechanisms on diverse soils and assessing its potential negative environmental impacts were the main goals of the research. The study has important ramifications for efficient pesticide management in agricultural and environmental settings since it addresses hitherto unreported factors such as sorption, hydrolysis, photolysis, and degradation.

## 2. Experimental Section

### 2.1. Materials

Analytical grade insecticide BF (C_23_H_22_ClF_3_O_2_) (99.9% pure) is purchased from Dr. Ehrenstorfer, Augsburg, Germany. Analytical grade acetone, methanol, calcium chloride (CaCl_2_·2H_2_O), and anhydrous sodium sulfate were utilized for experimental purposes. Stock solution of BF for adsorptive and degradative analysis was prepared in distilled water obtained from General laboratory, Fatima Jinnah Women University, Rawalpindi.

### 2.2. Instruments

Laboratory instruments used in this study include weighing balance (AUX220, Shimadzu company), shaker incubator (IRMECOS from Germany), UV-Vis Spectrophotometer (BMS-1602), Hot plate (MSH-20D, WiseStir manufacturer), and GCMS (GCMS-QP5050 from Shimadzu). The column details for gas chromatography-mass spectrometric analysis are 30 m × 0.250 mm ID fused silica, 0.25 μm column diameter, and Db-5. Split less injection system was used to inject sample at 250°C temperature with 1 min activation. The sample injected was about 1 μL in quantity.

### 2.3. Soil Sampling and Treatment for BF Assessment

Soil samples were prepared from 10 distinctive agricultural zones of Pakistan, with emphasis laid on such areas having the least use of agrochemicals. Soil samples were collected from 10 different regions in February 2023. Samples were obtained from 10- to 20-cm depth with augur, placed in polythene bags, and transferred to the laboratory for further analysis [24]. All samples were placed in greenhouse for drying and passed through a 2-mm mesh sieve for making it homogenized and stored in air-tight jars for further analysis.

Physicochemical analysis was performed for sample characterization, which includes soil pH determination, electrical conductivity (EC) measurement, identification of texture (percentage of sand, silt, and clay), OM content, salinity, total organic carbon (TOC), total nitrogen content, and heavy metal analysis.

### 2.4. BF Stock Solution and Dilution Preparation

Ten ppm stock solution of pyrethroid insecticide–BF was prepared by adding 10.35 mg of BF in 1000 mL of distilled water and stirred for 24 h. After the complete dissolution of insecticide, dilutions (0.25, 0.5, 0.75, 1, 2.5, 5, and 7.5 ppm) were prepared by adding respective amounts of stock solution and 10 mL of 0.1 M NaCl for electrolyte balance in 100-mL volumetric flasks. The flasks were filled up to the mark with distilled water for complete dilutions [25].

### 2.5. BF Sorption and Degradation Analysis

Batch equilibrium method was used for sorption analysis of BF as per OECD guidelines. Fourteen falcon tubes (15 mL each) including duplicates were used to analyze pesticide adsorption on soil samples. One control (i.e., pesticide solution) and experimental set (i.e., pesticide in NaCl solution and 5 g of soil) were used to avoid any contaminant participation and matrix effect. All falcon tubes were placed in an orbital shaker at 90 rpm for 24 h at room temperature. Next day, the tubes were centrifuged at 3500 rpm for 20 min followed by nylon filtration through 0.2-μm nylon filters. Clear aliquots of 1.5–3 mL were obtained for analysis through UV-visible spectrophotometer [25].

For desorption purposes, supernatant was discarded and tubes were reweighed. 9.5 mL of freshly prepared 0.1 M CaCl_2_ was added to the tubes and placed in shaker. Centrifugation was done after shaking followed by nylon filtration. Filtrate was analyzed by UV-visible for absorption spectrum, which is further used in isotherm drawing, linear and Freundlich model applications, and comparative sorption graphs.

Degradation of BF by soil inherent biodegradation and hydrolysis in soil is performed to study the degradative mechanism responsible for its partial disposition.

### 2.6. BF Hydrolysis in Soil

Hydrolysis is stated as breakdown in the presence of water molecules. BF hydrolytic mechanisms were studied by placing approximately 5 g of each soil in the volumetric flask and filling it with 100 mL of 10 ppm BF solution. These flasks were covered tightly by parafilm to avoid any environmental and microbial contamination and placed in incubator to maintain optimum conditions and uniform temperature of 27°C. The samples were taken out at uniform time intervals and extracted twice by using dichloromethane (2 mL + 2 mL). Anhydrous sodium sulfate (Na_2_SO_4_) was added to remove any moisture from the extract. Five extractions were performed after every 7-day interval and analyzed by UV-visible spectrophotometry. Furthermore, the extract was reduced to 1.25–1.5 mL by heating on a hot plate and storing in Eppendorf for analysis by GCMS.

### 2.7. Soil Inherent Biodegradation of BF

Degradation of BF by natural soil microbes is evaluated without application of artificial organic substances. BF degradation is observed in all 10 soil samples with intrinsic microbial fauna. In this analysis, no methanogenesis was done. Petri dishes were used to fill the soil samples, and 10 mL of pesticide solution was dispensed in each of them. The covered Petri dishes were then placed on greenhouse shelves to maintain natural light and temperature. Samples were extracted after regular time intervals by DCM and analyzed by UV–Vis for absorbance determination as time function and GCMS for identifying metabolites.

### 2.8. GCMS Conditions for BF

The prepared extracted samples were investigated by GCMS (QP5050, A, Shimadzu, Japan) system equipped with fused quartz capillary column of DB-5 MS (0.25 mm × 0.25 μm × 30 mm). The temperature program was customized as follows: Source temperature was 180°C and transfer line was set at 250°C. Injector temperature was preserved at 250°C. Helium was employed as carrier gas at gas flow rate of 1.20 mL·min^−1^.

### 2.9. Data Evaluation

Data analysis of BF sorption and degradation pathways was performed by using different mathematical equations. These equations are trailed by Naeem and coworkers [26].

Statistical examination (i.e., ANOVA and multivariate analysis; biplot treatment with principal component analysis) was executed for K_*d*_ and correlated soil physicochemical parameters with BF degradation rate, in MINTAB 17 (original statistical software, US).

## 3. Results and Discussion

### 3.1. Physicochemical Analysis of Soil Samples

Nature governs a variety of physical and chemical reactions at different rates in soil. However, soil as a receiving medium for many pollutants and contaminants from the atmosphere responds to this procedure in different ways. Pesticides are the major contaminants in the soil received during agricultural activities. Due to the continuous ingress and tenacity of pesticides in the food chain, their need should continuously be evaluated for their behavior so that they can be supervised and controlled in a manner that implies economic feasibility and sustainability [27]. In this context, complete pedospheric fate determination of BF is a fundamental step preceding to management. The present reported work evaluates the fate of insecticide BF through sorption and degradation via hydrolytic, photocatalytic, and soil-induced native reactions. Pesticide interacts with soil and behaves differently according to the soil physicochemical properties [28]. Therefore, investigations of soil physicochemical characteristics are inevitable. Prior to the understanding of BF fate, the carefully chosen soil samples were investigated for their physicochemical features as shown in Supporting Table 1.

Soil pH, salinity, and EC are analyzed by multimeter. Heavy metal analysis for each soil is performed by the acid digestion method followed by flame atomic absorption spectrometer (FAAS). Octagonal sieve shaker is utilized to determine the texture of each soil sample, while Walkley and Black method is utilized to calculate OM content.

The investigated physicochemical properties were soil pH, OM content, organic carbon (OC) content, EC, nitrogen content (N), saturation, and heavy metal analysis. The collected 10 soil samples exhibited variable physical and chemical properties. The effect of these properties on soil sorption and degradation was assessed.

OM ranges from 0.10% to 1.99% as shown in Supporting Table 1. The lowest percentage of OM is shown in the soils of Balochistan, Karak, and Kashmir and the highest in Parachinar (1.99%) and Jhang (1.48%). Jhang (Faisalabad division) is located in the northern area of Pakistan, so it receives heavy precipitation annually which can contribute to the efficient growth and better survival of soil inherent microbes [29]. This feature is then accordingly related to proper nutrients availability for microbial flora which enhances the OM formation. No proper and advanced treatment for wastewater exists in Balochistan and Kashmir. Therefore, heavy industries release their waste into adjoining water bodies, thus polluting surface and groundwater reservoirs, causing harm to biodiversity and decreasing crop productivity [30, 31].

Heavy metal detection is performed for all selected soil samples to analyze the contamination of pedospheric region by anthropogenic activities. Heavy metal includes all metallic entities having density greater than 5.0 g/cm^3^, and it is comprised of 45 elements. Heavy metals of soil constitute two groups according to their biochemical activity: One category is harmful to crops and includes Pb and Cd, while other one is beneficial to some extent in a limited range such as Cu, Ni, Zn, and Mn [32].

As heavy metal hinders the enzymatic activity of microbiota, soil with heavy concentration of heavy metals has very poor microbial activity. Heavy metal analysis (Mn, Cd, Cu, Ni, Pb, Zn, and Fe) of selected soils from all ecological zones of Pakistan was performed by FAAS. Lead (Pb) concentration is associated with automobile traffic activities [33], while zinc (Zn), copper (Cu), and nickel (Ni) are associated with industrial pollution and anthropogenic activities, respectively [34]. Lab experimental analysis of heavy metals is depicted in Supporting Table 1. The highest concentration of Pb is indicated in S5 (0.28 ppm) which might indicate the presence of M-15 motorway passing nearby. Zinc (Zn) is highest in S8 (Jhang) sample (6.8 ppm) due to heavy industrial effluent from cotton and sugar manufacturing plant [35]. Fe is indicated as the highest concentration in all samples. It might be an indicator of iron-enriched parent rock of the study areas. Variations in the presence of different concentrations of heavy metals are due to different geological conditions and anthropogenic activities. Additionally, Pakistani agriculture is primarily rain-fed and irrigation-based during the flood period. Such water may contain a heavy amount of suspended matter with heavy metals that may pollute the soil.

Textural analysis was performed by understanding the relative weight and abundance of sand, silt, and clay as indicated in Supporting Table 1. All of the sampling soils are loam, indicating a perfect balance in all soil constituents for plant growth [36]. EC ranged from 0.094 to 2.12 d Sm^−1^·s for Khairpur and Naushahro Feroze, respectively. EC is due to charge and particle development in soil and fluid components. EC is low for nonsaline soil while higher is for saline soil. Porosity enrichment of soil is another factor that contributes toward the EC of soil [37].

### 3.2. Batch Equilibrium Sorption Experiments of BF

Soil physicochemical analysis is performed to understand the soil particles' attachment to BF. Sorption experiments were performed by batch equilibrium method [38–40]. The multilayered adsorptive pattern is further analyzed by plotting in linear and Freundlich models. The best regression values were proved to be completely fitted in these models.

#### 3.2.1. Comparative Linear Isotherm for BF Insecticide

Soil physicochemical properties govern the shifting of BF insecticide peak. These properties alter the BF binding with soil particles which may change the sorption values. BF adsorption analysis was conducted by UV-visible spectroscopy, and comparative regression analysis was performed by plotting charts between equilibrium concentration and BF adsorption.

**Figure 1:**
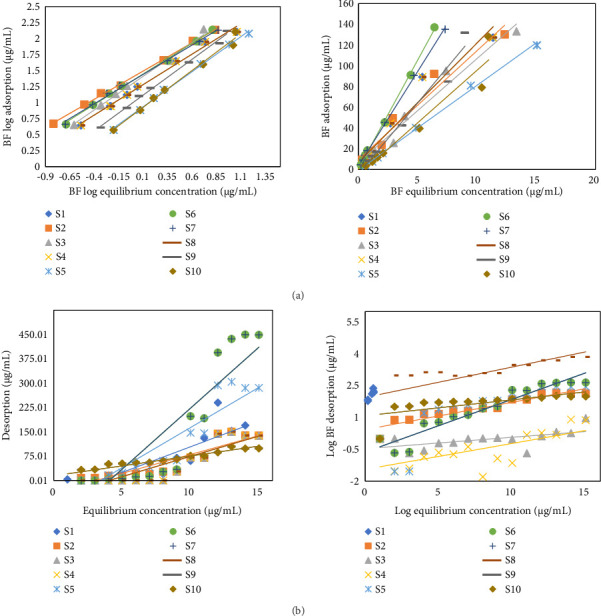
Comparative linear and Freundlich graphical representation of BF for (a) adsorption of BF in soils and (b) desorption of BF in soils.

Different parameters were investigated and analyzed in BF linear adsorption: soil adsorption coefficient (*K*_*d*(ads)_), K_om_, and K_oc_, which indicated the mobility of BF in soil with adsorptive interaction as depicted in Table 1 and Figure 1. The adsorption coefficient values (K_*d*_) varied from 7.27 to 25.89 μg·mL^−1^ with *R*^2^ values 0.90 to 0.99. It depicted strong and higher rate of adsorption resulted in hysteresis effect. These values exhibited the best fit in linear model of adsorption. The highest K_*d*_ value (25.89 μg/mL) is attained for S6 sample (Morro) showing maximum adsorption while the lowest value is depicted by S1 (7.27 μg/mL). The high adsorption percentage for BF is responsible for its low leaching. Rashid and coworkers worked for BF termiticidal leaching via column and obtained results with least leaching property and high degree of adsorption [41, 42]. Mobility or leaching index is utilized to list down or to categorize the BF as immobile. Mobility index is based on K_oc_ values. If these values are less than 50, it depicts higher mobility while 150 to 400 exhibited low mobility [43]. The highest K_oc_ value of 4991.53 μg·mL^−1^ is demonstrated by S10 (Dadyal) having least mobility; however, its adsorption is not high due to low OM content and high pH. Lowest K_oc_ values are shown by S9 (Dera Bugti) with low OM rendering to have more potential for draining. However, complete scenario for BF K_oc_ values exhibited low mobility and high adsorption trend. The adsorption distribution coefficient depicted best correlation with OC and soil OM content proving to be a strong controlling factor for adsorption [44].

BF demonstrated “C” type isothermal curve. It is depicted that by increasing concentration of BF, adsorption values increase to a reasonable extent. BF portrayed fluctuating adsorption values due to variable physicochemical properties. Values for K_d_ showed the following order of adsorption in the present research:

(1) $$\begin{array}{c} \left(S6>S4>S7>S2>S3>S8>S9>S10>S5>S1\right)\ldots \left(\mu g/\text{mL}\right). \end{array}$$

The thermodynamic parameter of adsorption is also assessed for BF insecticide. Δ*G* (i.e., Gibbs free energy) were calculated which were less than −40 kJ/mol. It depicted the physisorption interaction and exothermic reaction of BF with soil. In the present study, Δ*G* values ranged from −12.1 to −22.27 kJ·mol^−1^ which corresponds to the presence of weak Van der Waal forces of attraction between pesticide and soil.

#### 3.2.2. Comparative Freundlich Isothermal Analysis for BF Insecticide

Freundlich isotherm for BF displayed distribution coefficient (K_f_) ranges from 1.35 to 25.06 kJ·mol^−1^. Value of K_f_ corresponds directly to the soil's OM. The higher the OM value of soil, the greater the K_f_ value. However, some exceptions are present in these data due to the influence of other soil's physicochemical properties such as pH. Value of *R*^2^ is between 0.90 and 0.98. The recent study has displayed a close correlation between soil physicochemical properties and adsorption rate. S6 sample showed the highest adsorption value (i.e., K_d_ = 25.89 μg/mL; K_f_ = 26.06 μg/mL) because of its higher OM content (1.99%), which facilitated its stronger binding to the soil particles leading to a higher rate of adsorption [26].

#### 3.2.3. Comparative Analysis for Desorption Isotherm

BF desorption analysis is performed to determine the desorption coefficient constant. Soil is a recipient of different contaminating agents from anthropogenic activities. However, soil itself can act in such a mechanistic way which can detox the pedospheric compartment to an appreciable extent. One such process is categorized as the desorption phenomena. This detachment of contaminant particles from soil is however dependent on forces involved in adsorption process and soil physicochemical parameters. UV-visible analysis is performed to determine the concentration of detached BF. Further calculations are performed, and graphs were plotted between equilibrium concentration and BF desorbed (Figure 1(b)).

C-type isotherms are observed for all selected samples. Desorption is the reverse phenomena of adsorption; however, it also correlates with soil's individual physicochemical properties. For instance, high pH will increase the desorption rate to a reasonable extent. *K*_*d*(*de* *s*)_ values ranged between 1.78 and 1269 μg/mL. Soil 10 demonstrated the highest desorption (i.e., 1269 μg/mL), while the lowest desorption is shown by S2 (i.e., 1.78 μg/mL). The fact behind this trend is high pH and clay content, low OM, and electronegativity which increases the desorption rate. Trend of desorption values in the selected samples is as follows:

(2) $$\begin{array}{c} S2<S4<S3<S5<S8<S6<S7<S9<S1<S10. \end{array}$$

Linear and Freundlich models were assessed for BF desorption analysis. *R*^2^ for linear desorption values reached to 0.99 which proved it to be the best fit model for BF desorptive analysis (Table 2).

**Figure 2:**
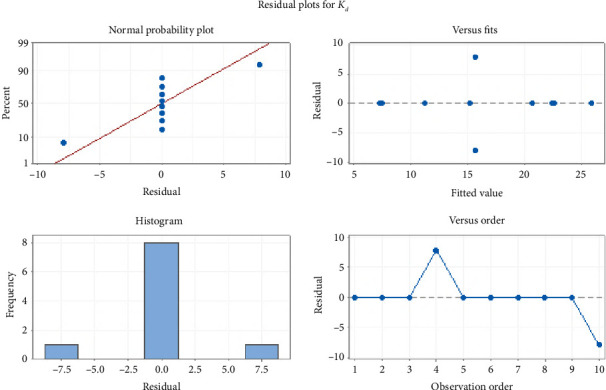
MINITAB-driven arithmetical significance assessment through residual plots for *K*_*d*_ of BF with physiochemical parameters.

The proportion between *n*_(ads)_ and *n*_(des)_ is known as apparent hysteresis phenomena which depicted the irreversibility of adsorption in the selected samples. Adsorption–desorption phenomena of BF were also evaluated by MANOVA (i.e., multivariate analysis of variance) as depicted in Figure 2. MANOVA was used to compare soil significant physical and chemical parameters such as pH, TOC, OM, and TOC with varying distinguishable factor such as adsorption parameter K_d_. An extensive significance was depicted by the best fit of results as reflected by residual plots. Moreover, regular normalized behavior was articulated by residual plots for sorption of BF in soil samples.

### 3.3. Degradative Mechanistic Analysis of BF

#### 3.3.1. Hydrolytic Degradation

Hydrolysis is taking place in pedosphere along with many other reactions in an innate manner. The hydrolytic mechanism is considered in both organic and electrical substances for a wide range of pesticides [45, 46].

**Figure 3:**
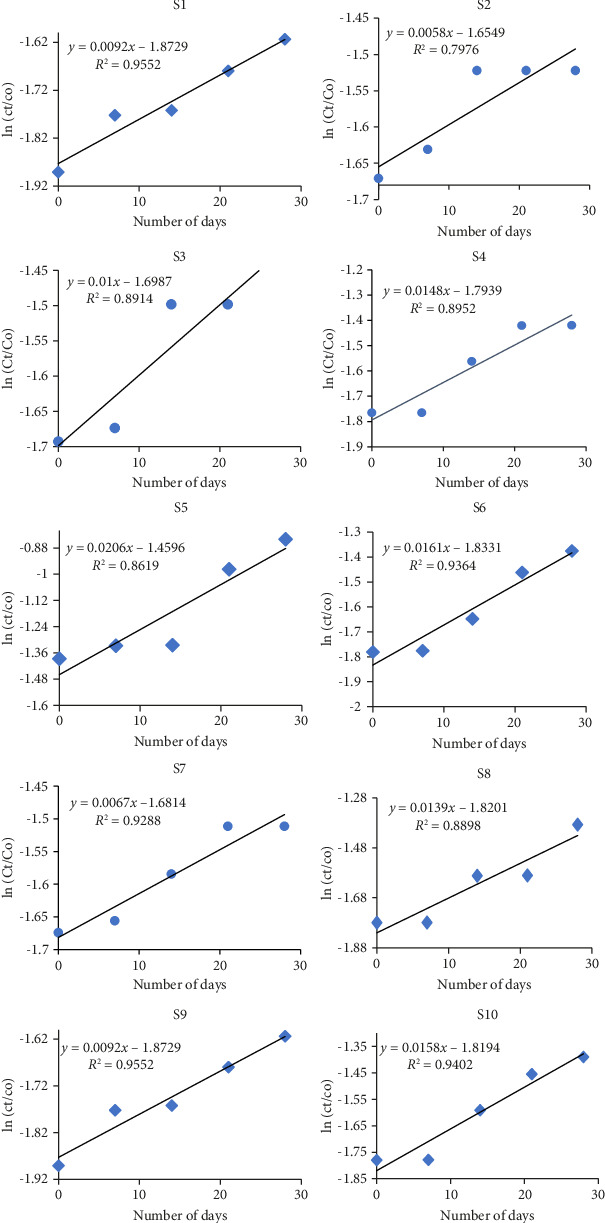
UV-visible-based half-life and percentage degradation of BF by hydrolysis.

Hydrolysis usually follows the first-order reaction kinetics, in which rate of pesticide hydrolysis is directly related to concentration of pesticide. As the half-life of pesticide is independent of its concentration [47], so in order to understand the rate of pesticide hydrolysis in simulated lab-based experimental conditions, it is being proposed by many research types that high concentration of pesticide is needed for understanding the hydrolytic behavior by removing procedural complexity, arduousness [48, 49], and to enhance the reliability of results in compliance to the natural hydrolytic degradation of pesticide. Degradation percentage for S4 was observed as 83% (Table 3) due to the presence of high sand content and low OM. Heavy quantity of sand in composition of soil makes it difficult and obstructs the binding of pesticide with soil, resulting in availability of more pesticide molecules to hydrolyze. In addition, low pH of S4 makes it acidic in nature and in acidic medium pesticide is more prone to degradation through hydrolysis. Meena and coworkers reported in a recent study that BF depicted lowest persistence in acidic medium [50], while the minimum degradation percentage was depicted by S3 (i.e., 9%) as shown in Figure 3. Low rate of degradation in S3 might be attributed as its lower OM and sand content. Highest OM was possessed by S6 and S7 which might be attributed as decrease in hydrolysis. It has been testified and stated that BF has a long half-life (*t*_1/2_ = 251–1950 days) in watercourse sediment [51].

Most of the pyrethroid insecticides including BF can be easily hydrolyzed by enzyme esterase trailed by immediate oxidation by cytochrome P450 [52]. However, slow hydrolysis takes place in aquatic species like fish because of hydrolase lake [53]. Half-life of BF in soil-based degradative mechanisms ranges from 2 weeks to 1 year, which depends upon soil texture, pH, TOC, moisture content, and other conditions [54–56]. Major metabolite of BF by hydrolysis is 4-hydroxy BF [57]:

(3) $$\begin{array}{c} C_{23}H_{22}C1F_{3}O_{2}+H_{2}O⟶C_{23}H_{22}C1F_{3}O_{3}+{\text{OH}}^{-1}. \end{array}$$

Various soils are used for the evaluation of hydrolytic phenomena since a variety of findings and trends are obtained in this experiment, and thus, attainment of a conclusive statement for the BF hydrolysis is difficult.

#### 3.3.2. BF Soil-Based Degradation

Pedospheric zone of environment undergoes many physicochemical changes due to microbial activities of soil microbiota. Synthetic chemicals applied for beneficial purposes on soil may undergo different pathways and are modified into daughter products.

It has been reported that chemico-physical methods like sorption [58, 59], photolysis [60], and hydrolysis [61] are predominantly utilized methods to transform or degrade the synthetic chemicals [62]. However, such methods are responsible for the production of more toxic intermediates and thus are more expensive in nature [63]. In contrast to these chemico-physical methods, biological-based methods are considered as more cost-effective, higher efficacy, and environmentally benign approach.

**Figure 4:**
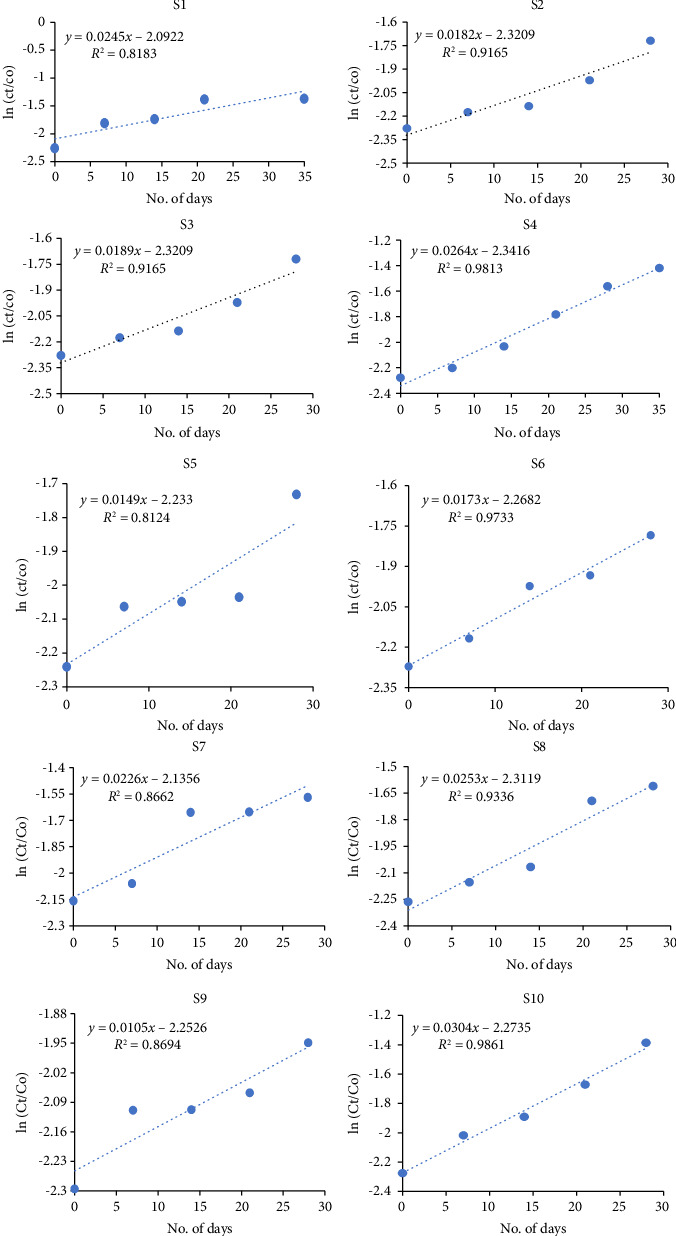
Graphical representation of interaction between BF and soil microbiota.

Soil microbial community utilizes their specific intrinsic characteristics to remediate the pollutant at a faster rate. It resulted in the complete disintegration of the contaminant into CO_2_ and water (H_2_O) without any toxic and harmful intermediate products. For effective remediation, enzymes of microorganisms must attack the contaminant and transform it into less toxic agents [64]. However, soil humidity, moisture, and temperature fluctuation should be in consideration as it can alter the whole chemico-physical makeup of pedosphere [65]. Harsh temperature also affects the soil microbial flora. Microbiota of soil include algae, fungi, actinomycetes, bacteria, and nematodes [66]. Microbial degradation may be determined to fulfill energy demands of microbiota, or need to clean the environment, or cometabolism. The pervasive nature of microbial community, their large number, and biomass as compared to other living beings on earth, variable catalytic activity and capability [67], and their genetics, and their capability to work even in the extreme environmental conditions makes them to be useful as bioremediating agent [68]. BF degradation in soil might be difficult as soil's OM may hinder the possible degradation by binding to the BF molecules (Table 4; Figure 4). However, soil's natural and innate microbiota can efficiently act as detoxifying agent to remediate the pedospheric environment.

In the present study, highest degradation by soil inherent microbes is observed in S10 (60%) which might be attributed as its high pH and low OM content. Lowest degradation is depicted in S9 due to lower annual precipitation level in Balochistan region which in turn is responsible for its lower OM and poor soil biota. The predicted change in the composition of dissolved OM is a result of ongoing global climate warming [69].

**Figure 5:**
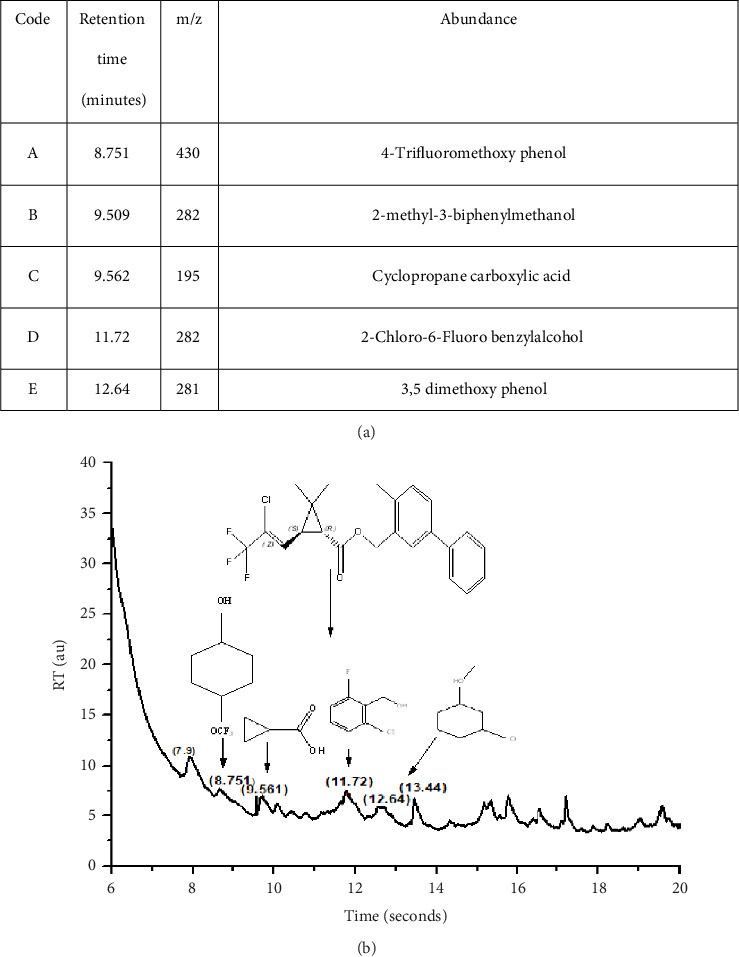
(a) GCMS products and other parameters matched and listed with NIST library. (b) GCMS-based disintegration pattern of BF and chromatogram for soil inherent degradation of sample.

GCMS investigation was executed on the highest degradation of final extraction to elucidate the metabolic products as depicted in Figure 5. GCMS revealed the five major BF degradation products which can be attributed to its metabolites. The MS peak at retention time 8.751 corresponds to 4-trifluoromethoxy phenol, while retention times 9.509, 9.562, 11.72, and 12.64 match to 2-methyl-3-biphenyl methanol, cyclopropane carboxylic acid, 2-chloro-6-fluoro benzyl alcohol, and 3,5 dimethoxy phenol, respectively, as presented by NIST library.

**Figure 6:**
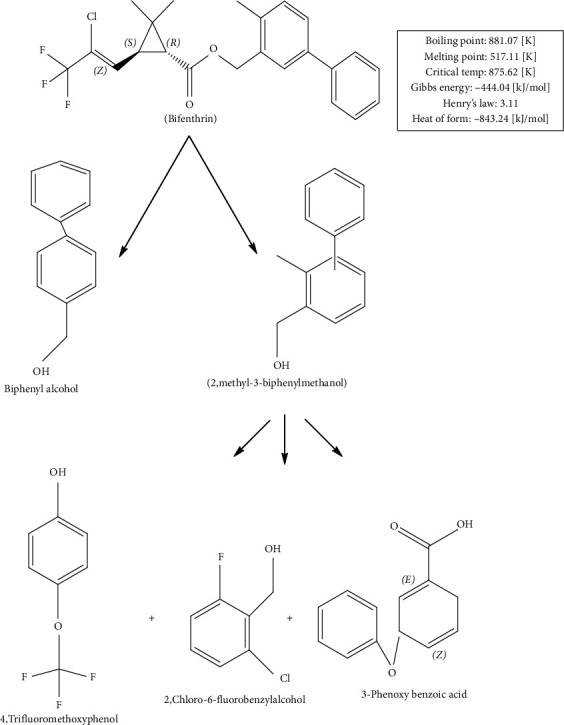
Proposed metabolic degradation pathway of bifenthrin.

On the basis of these metabolites, degradation pathway of BF can eventually be proposed (Figure 6). The parent molecule of BF is first metabolized by hydrolase to biphenyl alcohol and 2-methyl-3-biphenyl methanol. Afterward, 2-methyl-3-biphenyl methanol is transformed to 4-trifluoromethoxyphenol, 3-phenoxybenzoic acid, and 2-chloro-6-fluorobenzyl alcohol by biphenyl cleavage. Such products are obtained from reductive dichlorination and oxidative reactions (cytochrome P450s) [53].

#### 3.3.3. BF Photophysical Deterioration

Photolysis is the breakdown of chemical moieties in the presence of sunlight. It excites the electronic setup of chemical specie to an excited state by absorbing photon and results in collapsing of whole structure.

**Figure 7:**
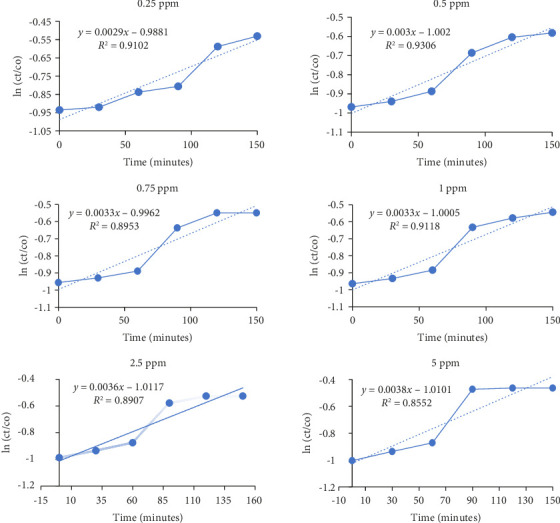
UV-visible originated half-life and photocatalytic degradation of BF.

Photodegradation of BF is performed by utilizing dilutions of different concentrations, i.e., 0 ppm, 0.25 ppm, 0.5 ppm, 0.75 ppm, 1 ppm, 2.5 ppm, and 7 ppm. All of these dilutions were prepared in distilled water from BF stock solution. Two batches of dilutions were prepared. Control batch is placed in dark, while experimental setup is analyzed after putting in direct sunlight from 12 p.m. to onward. UV-visible spectrophotometer is utilized to analyze and understand the effect of sunlight on BF (Figure 7). Experimental results showed that BF degrades to almost negligible extent in dark conditions which confirms its zero percent self-decay. However, sunlight-driven degradation is observed in experimental batch. Absorbance maxima were recorded, and wavelength was monitored by UV-visible spectrophotometer. The reaction pathway and kinetics were estimated and plotted against (ln (Ct/Co)). Photo-chemical decay of BF followed first-order reaction kinetics.

**Figure 8:**
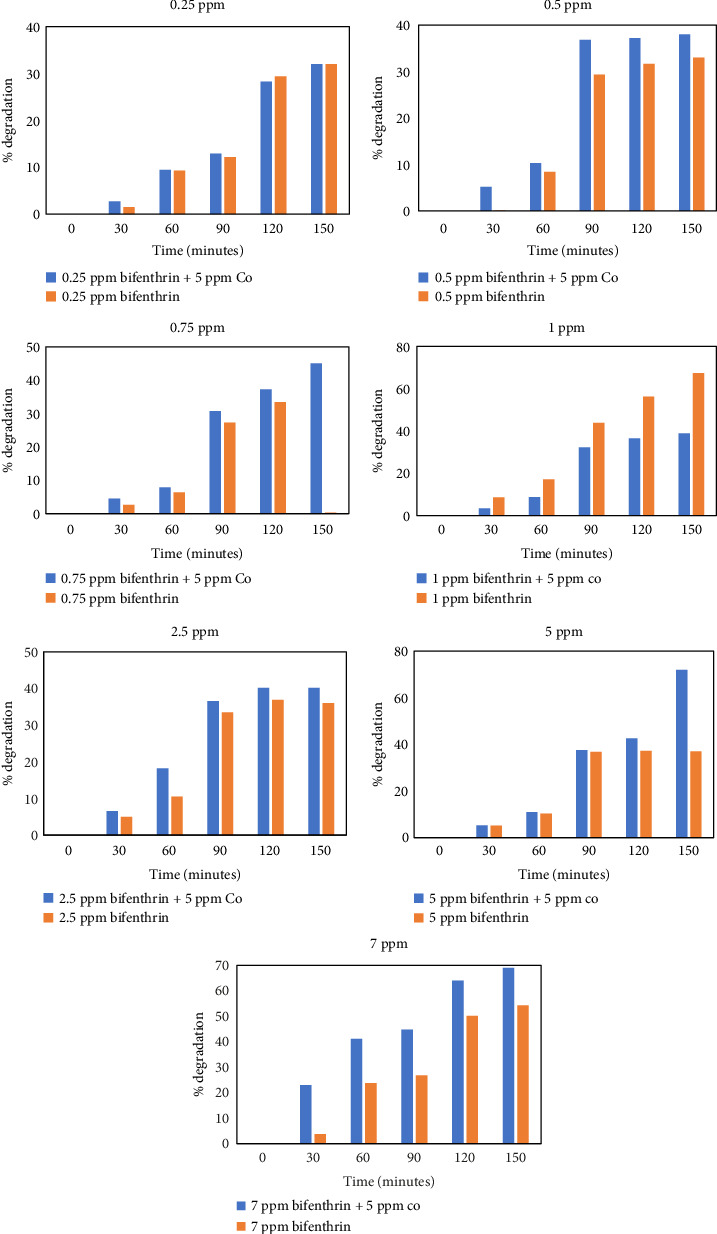
Comparative degradative analysis of BF with and without Co addition.

Highest photolytic rate constant (*k*) and minimum half-life (*t*_1/2_) in the present study are detected of concentrations 7 mg/L (i.e., 7 ppm) 0.0057 and 121.5 days, respectively (Table 5). Similar experiments were performed by adding 5 ppm Co (i.e., 5 mg/L) in BF dilutions. As a known fact, cobalt can act as an efficient photocatalyst [70] which enhanced the photodegradation of BF by absorbing extra photons and transferring the energy to BF for catalytic breakdown (Figure 8). Comparison of BF degradation under various conditions in the literature is provided in Table 6:

(4) $$\begin{array}{c} \text{Photoatalyst}\left(\text{Cu}\right)+\text{hv}⟶e\left(\text{cond}.B\right)+h^{+}\left(\text{Valence}.B\right)\\ e\left(\text{cond}.B\right)+\text{Oxygen}⟶{\text{Oxygen}}^{-}\\ {\text{Oxygen}}^{-}+e\left(\text{cond}.B\right)+2H^{+}⟶{\text{OH}}^{\cdot }+{\text{OH}}^{-}\\ \text{BF}\left(\text{contaminat}\right)+{\text{OH}}^{\cdot }\overset{0–9^{{}^{\circ}}}{⟶}\frac{\text{metabolites}}{\text{degradation products}} \end{array}$$

## 4. Conclusion

The present study successfully investigated the adsorption/desorption behavior of BF and its degradation mechanisms, via hydrolysis, photolysis, and microbial breakdown in chosen soil samples from different agricultural Asian zones. The distribution coefficient (K_d_) for BF ranged from 7.27 to 25.89 μg/mL, indicating a moderate binding affinity and stability in the soil, while *R*^2^ values ranged from 0.92 to 0.99. Since BF is less likely to contaminate groundwater when used within permitted limits, its adsorption in soils—presumably caused by physisorption or van der Waals forces—indicates less leaching potential and mobility. The half-lives of the hydrolysis, soil-induced biodegradation, and photolysis processes were 13.5 days, 12 days, and 121.5 days, respectively. Notably, the S10 sample showed the highest degradation (60%) due to the presence of natural soil microbes. The Gibbs free energy (ΔG) values ranging from −22.27 and −12.10 kJ/mol suggest van der Waals forces and an exothermic, spontaneous reaction. Additionally, based on GCMS analysis of degradation products, a possible pathway for BF degradation was suggested, emphasizing the possible advantage of adding outside agents to speed up deterioration. The study also showed that the rate of photocatalytic breakdown of BF rose from 54% to 69% with the inclusion of cobalt, a strong photon absorber, as a photocatalyst. These findings advance our knowledge of BF behavior in soils and its vulnerability to diverse degradation processes, assisting researchers, environmentalists, and botanists in their investigation of toxins in various environmental niches. According to the tenets of green chemistry, the methods and approaches used in this study support the creation of ecologically friendly pest management practices and the mitigation of pesticide-induced environmental deterioration.

## Figures and Tables

**Table 1:** Linear and Freundlich adsorption of BF on 10 diverse soils.

Sr. no	Soil sample	Linear adsorption model values				Δ*G* (kJ·mol^−1^)	Freundlich adsorption model values			
		*K*_*d*(ads)_ (μg/mL)	K_oc_ (μg/mL)	K_om_ (μg/mL)	*R* ^2^		*K*_*f*_ (μg/mL)	K_foc_ (μg/mL)	*n*_*a*_	*R* ^2^
S1	Khairpur	7.27	4065	7.76	0.93	−12.10	22.06	7120.76	1.15	0.96
S2	Morro	22.40	2129	7.12	0.95	−17.64	1.35	128.42	1.09	0.98
S3	Naushahro Feroze	20.69	2973	7.45	0.94	−18.46	20.32	2920.11	0.95	0.92
S4	Karak	23.65	1516	9.07	0.98	−14.30	25.55	15,096.15	1.12	0.94
S5	Hazara	7.505	1486	6.76	0.98	−16.74	6.53	1294.85	0.89	0.97
S6	Parachinar	25.89	4162	7.79	0.99	−20.20	26.06	4104.01	1.13	0.98
S7	Kurram	22.59	1955	7.03	0.99	−19.20	22.64	1958.99	1.04	0.96
S8	Jhang	15.20	1768	6.93	0.95	−17.18	15.45	1796.86	1.05	0.90
S9	Dera Bugti	11.23	13,872	8.99	0.97	−22.27	10.18	12,571.31	0.89	0.95
S10	Dadyal	7.78	4991	7.96	0.92	−19.73	6.53	4191.66	0.67	0.90

**Table 2:** Linear and Freundlich desorption of BF on 10 soil samples.

#	Sample	*K*_*d*(des)_ (μg/mL)	*K*_*f*_ (μg/mL)	*n*_*d*_	*H*
S1	Khairpur	33.76	33.10	1.75	0.65
S2	Morro	1.78	21.62	1.01	0.90
S3	Naushahro Feroze	5.14	2.69	1.44	1.51
S4	Karak	2.43	1.60	0.98	0.87
S5	Hazara	17.84	5.82	0.57	1.56
S6	Parachinar	19.80	16.90	1.44	1.37
S7	Kurram	20.01	15.60	1.05	0.99
S8	Jhang	18.90	3.60	0.79	1.14
S9	Dera Bugti	20.83	16.92	0.84	0.93
S10	Dadyal	20.03	19.9	0.99	1.03

**Table 3:** Hydrolysis degradative parameters of BF in 10 soil samples.

No	Region	Hydrolytic rate constant (*k*)	Half-life (*t*_1/2_) (days)	Percentage degradation (%)
S1	Khairpur	0.09	76	24
S2	Morro	0.05	119.4	13
S3	Naushahro Feroze	0.01	223.5	9
S4	Karak	0.02	13.5	83
S5	Hazara	0.01	46.8	29
S6	Parachinar	0.01	43.0	33
S7	Kurram	0.00	103.4	15
S8	Jhang	0.01	49.8	32
S9	Dera Bugti	0.09	76.1	24
S10	Dadyal	0.01	43.8	32

**Table 4:** Soil inherent biodegradation parameters of BF.

No.	Region	Soil-based rate constant (*k*)	Half-life (*t*_1/2_) (days)	Percentage degradation (%)
S1	Khairpur	0.0245	12	58
S2	Morro	0.0182	29.2	43
S3	Naushahro Feroze	0.0189	36.6	43
S4	Karak	0.0264	26	57
S5	Hazara	0.0149	46.5	39
S6	Parachinar	0.0173	40	38
S7	Kurram	0.0226	30.6	44
S8	Jhang	0.0253	27.3	48
S9	Dera Bugti	0.0105	66	29
S10	Dadyal	0.0304	22.7	60

**Table 5:** Photocatalytic disintegration parameters of BF insecticide.

Dilutions	Photolysis rate constant (*k*)	Half-life (*t*_1/2_) (days)	Percentage degradation (%)	Percentage degradation with Co 5 mg/L or 5 ppm (%)
0.25	0.0027	238.96	32	32
0.5	0.003	231	33	38
0.75	0.0033	210	33	40
1	0.0033	210	34	45
2.5	0.0036	192.5	36	59
5	0.0038	182.36	37	59
7.5	0.0057	121.57	54	69

**Table 6:** Comparison of bifenthrin degradation under various conditions in the literature.

Sr. no	Degradation mode	Bifenthrin used/degradation percentage	Conditions	Half-lives (*t*_1/2_)	References
1	Soil inherent	0.1 mg/kg	Clayey soil	21.2	[71]
2	Photolysis	41%	24-h UV irradiation	—	[72]
3	Biodegradation by *Candida pelliculosa*	50 mg/L degraded completely	32.3°C, 7.2 pH	4-days	[73]
4	Biodegradation by *Bacillus* species	10 mg/L degraded to 93%	32°C, 130 rpm	—	[74]

## Data Availability

Data will be made available on demand from authors.

## Supporting Information

A file labeled as Supporting Information is attached with main manuscript containing a table provided with physicochemical parameters of heterogonous soil samples collected from different geographical zones.
