# Supplementary material for: Bifenthrin's Environmental Fate: An Insight Into Its Soil Sorption and Degradation Studies
Source: J Anal Methods Chem. 2024 Nov 25;2024:8868954. doi: 10.1155/jamc/8868954 (PMC11614517; doi:10.1155/jamc/8868954)
Supplement: Supporting Information — Additional supporting information can be found online in the Supporting Information section. [file 8868954.f1.docx]

**Supplementary material**

**Bifenthrin's Environmental Fate: An insight into its soil sorption and degradation studies**

**Supplementary Table 1.** **Physicochemical parameters of heterogonous soil samples collected from different geographical zones in a random sampling mode**

| **# Sample** | | **pH** | **OM** | **Saturation** | **Salinity** | **Texture** | **Texture Classes** | | | **EC**  **(dSm^-1^)** | **TOC %** | **TN%** | **Mn**  **ppm** | **Cd**  **ppm** | **Cu**  **ppm** | **Pb**  **ppm** | **Zn**  **ppm** | **Fe**  **ppm** |
| --- | --- | --- | --- | --- | --- | --- | --- | --- | --- | --- | --- | --- | --- | --- | --- | --- | --- | --- |
|  |  |  |  |  | % |  | Sand | Silt | Clay |  |  |  |  |  |  |  |  |  |
| 1 | Khair Pur | 8.60 | 0.63 | 42 | 0.1 | Loam | 42 | 41 | 17 | 0.094 | 0.366 | 0.58 | 3.88 | 0.27 | 0.27 | 0.17 | 3.53 | 54.7 |
| 2 | Morro | 8.727 | 1.07 | 43 | 0.7 | Loam | 40 | 40 | 20 | 1.66 | 0.622 | 1.02 | 4.11 | 0.22 | 0.52 | 0.18 | 4.79 | 54.8 |
| 3 | Noshero Feroz | 8.584 | 1.20 | 42 | 1.5 | Loam | 31 | 36 | 23 | 2.12 | 0.696 | 1.15 | 5.34 | 0.16 | 0.43 | 0.19 | 4.48 | 55.3 |
| 4 | Karak | 8.742 | 0.27 | 41 | 0.7 | Loam | 42 | 38 | 20 | 1.78 | 0.156 | 0.22 | 2.68 | 0.15 | 0.45 | 0.16 | 4.64 | 53.7 |
| 5 | Hazara | 8.674 | 0.87 | 40 | 0.2 | Loam | 36 | 42 | 22 | 0.99 | 0.505 | 0.82 | 3.48 | 0.15 | 0.69 | 0.28 | 5.52 | 54.3 |
| 6 | Parachinar | 8.690 | 1.99 | 42 | 0.2 | Loam | 42 | 45 | 13 | 1.00 | 1.156 | 1.94 | 4.82 | 0.16 | 0.80 | 0.23 | 5.55 | 55.5 |
| 7 | Kurram | 8.593 | 1.81 | 46 | 0.1 | Clay Loam | 38 | 34 | 28 | 1.00 | 1.052 | 1.76 | 4.49 | 0.17 | 0.52 | 0.24 | 5.09 | 55.6 |
| 8 | Jhang | 8.576 | 1.48 | 43 | 0.3 | loam | 39 | 36 | 25 | 1.72 | 0.860 | 1.43 | 4.19 | 0.21 | 1.77 | 0.23 | 6.8 | 54.6 |
| 9) | Dera Bugti | 8.584 | 0.14 | 38 | 0.2 | Loam | 39 | 42 | 19 | 1.77 | 0.081 | 0.09 | 2.58 | 0.15 | 0.30 | 0.09 | 2.92 | 50.8 |
| 10 | Dadyal | 9.078 | 0.27 | 37 | 0.2 | loam | 38 | 46 | 16 | 0.72 | 0.156 | 0.22 | 4.01 | 0.15 | 0.17 | 0.13 | 2.77 | 54.4 |
